# Supplementary material for: A transient reversal of miRNA-mediated repression controls macrophage activation
Source: EMBO Rep. 2013 Sep 13;14(11):1008–16. doi: 10.1038/embor.2013.149 (PMC3851954; doi:10.1038/embor.2013.149)
Supplement: Supplementary Information [file embor2013149s1.pdf]

Supplementary Materials and Methods

Cell Culture and Reagents Primary murine peritoneal macrophages were elicited by 4% starch. A total of 1.5 ml of the solution was injected i.p. in Balb/C mice and peritoneal macrophages were isolated by lavage of the peritoneal cavity with RPMI. Cells were then cultured in RPMI 1640 supplemented with 2 mM L-glutamine and 10% heat-inactivated fetal calf serum (FCS). 10 μg/ml α-Amanitin (Calbiochem, La Jolla, CA) or 50 ng/ml Rapamycin (Calbiochem, La Jolla, CA) was used to stop *de novo* transcription and to slow down translation respectively. DMSO was added to controls (final concentration of 0.1%). LPS from *Salmonella typhimurium* (#L6143) was from Sigma-Aldrich. PMA was from Calbiochem and CpG was from IDT DNA. p38 MAPK Inhibitor (SB203580), ERK Inhibitor (PD98059) JNK Inhibitor (SP600125) were from Calbiochem.

Transfection of RAW 264.7 macrophages Transfections of RL or FL reporter plasmids were performed using Lipofectamine 2000 (Invitrogen) following manufacturer's protocol. siRNAs were transfected at 100 nM concentration using Lipofectamine 2000 (Invitrogen). Transfection of HA-Ago2, N-HA-Ago2, N-HA-Ago3, FH-Ago2, FH-Ago2Y529F or FH-Ago2Y529E was done with Fugene HD (Roche) according to manufacturer's protocol. 2'-O-Me-let-7a or 2'-O-Me-miR122 was transfected at 100 nM concentration using Fugene HD (Roche). For mRNA transfections, cells were transfected with 50 ng reporter mRNA in 24 well format using Lipofectamine 2000 (Invitrogen) and luciferase activity measured 16 h post-transfection.

Luciferase Assay RL and FL values were measured using Dual-luciferase Assay kit (Promega) following product manual on a VictorX3 plate reader (Perkin Elmer).

Mice Adult Balb/C mice were obtained from the animal facility of CSIR-Indian Institute of Chemical Biology and all the experimentations were performed according to the National Regulatory Guidelines issued by the Committee for the Purpose of Supervision of Experiments on Animals, Ministry of Environment and Forest, Govt. of India.

Parasite Infection to RAW 264.7 cells *Leishmnia donovani* strain AG83 (MAOM/IN/1083/AG83), was originally obtained from an Indian VL ("kala-azar") patient and maintained in golden hamster. 2nd - 4th passage cultures of *L. donovani* promastigotes were used to infect RAW 264.7 cells in 10:1 ratio for all experiments.

Western Blotting Imaging of all western blots was performed using an UVP BioImager 600 system equipped with VisionWorks Life Science software (UVP) V6.80. A detail list of antibodies is given in table S2.

RNA Isolation and miRNA or mRNA Detection Total RNA was extracted by using the TRIzol reagent (Invitrogen) according to the manufacturer's protocol. 10 μg of total RNA was electrophoretically separated on a 15% 7.5 M Urea-PAGE, transferred to a Nylon NY+ membrane (Millipore), cross linked and hybridized with ^{32}P-labeled 22 nt anti-sense DNA probes specific for respective miRNAs or U6 snRNA. Phosphorimaging of the blots was performed in Cyclone Plus Storage Phosphor System (Perkin Elmer). For quantification of miRNAs, assays were also performed by using the TaqMan® Universal PCR Master Mix (Applied Biosystems) following the manufacturer's instructions.

For mRNA quantification, total RNA was used to prepare cDNA with random nonamers (Eurogentec Reverse Transcriptase Core Kit) and the produced cDNA was used for PCR amplification with gene specific primers with MESA GREEN qPCR Master Mix Plus for SYBR Assay with Low Rox (Eurogentec). The comparative C_t method, which typically included normalization by the 18S rRNA levels for each sample, was used for relative quantitation. Real-Time analysis was performed on a 7500 Real-Time PCR system (Applied Biosystems). The primer sequences are given in the table S1 below.

Affinity purification of miRISC and cleavage assay FLAG-HA-Ago2, FH-Ago2 or FH-Ago2Y529F expression plasmid was co-transfected in RAW 264.7 cells with plasmid expressing pre-miR122. Cell lysis and miRISC purification was done following the published

protocols [1] . Affinity purified miRISC-122 was assayed for target RNA cleavage using a 36 nt RNA 5'AAAUUCAAACACCAUUGUCACACUCCACCAGAUUAA3' bearing the sequence complementary to mature miR-122 (underlined). Target RNA cleavage assays were carried out in a total volume of 30μl with 10 fmoles of 5' ^{32}P-labeled RNA and protein equivalent amounts of RISC at 30°C for 30min. RNA was isolated and cleaved products were analyzed on a 12% denaturing 8M Urea- PAGE followed by autoradiography.

Plasmids and Oligos Description of plasmids pRL-3xbulge-let-7a, pRL-per-let-7a, pRL-Con, pFL and N-HA-Ago3 were all published previously [2] and were the kind gifts from Witold Filipowicz. The pRL-3xBulge-miR-122 reporter contains three sites with a sequence AUACUAUUGUUAACACACACUCCA. Upon base-pairing with miR-122, this sequence forms a duplex containing a bulge in its central region. FH-Ago2, FH-Ago2Y529F, FH-Ago2Y529E plasmids were also described earlier [3] and FH-Ago1 described in [4] and were kind gifts from Gunter Meister. All the Argonaute plasmids contain human Ago sequence and were used for transfection in RAW 264.7 cells. GFP reporter plasmids were generated by replacing RL encoding sequence of pRL-Con and pRL-3xBulgeB-let-7a with GFP encoding fragment cloned in the NheI, XbaI site. A plasmid encoding precursor miR-122 was generated by inserting the PCR amplified fragment encoding the pre-miR-122 under a U6 promoter as described previously [5].

Primers HMGA2FP-5'-GCTCTAGATACTAATAGTTTGTTGATCTG-3 and HMGA2RP-5'-ATAAGAATGCGGCCGCGACCAAACTTTATTACTCATT-3' were used to amplify the HMGA2 3'UTR fragments from the parent plasmids described elsewhere [6] that were subsequently cloned into the XbaI and NotI sites of pRL-Con plasmid for construction of RL-HMGA2 plasmid. Similarly TLR4 3'UTR was cloned using FP 5'-GCTCTAGAGCCGCCAGTGTGCTGG-3' and RP 5'-ATGCTCGAGCGGCCGCCA-3' into RL reporter. *p27* 5'UTRFP-5'-GCGCGCTAGCCTTCTTCGTC-3' and *p27* 5'UTRRP-5'-CCGGGCTAGCCTTTCTCCC-3' were used to amplify and clone the 472 bp product of the full length Cdk inhibitor 1B (*Kip1, CDKN1B, p27*) 5'UTR in pRL-Con and pRL-3xbulgeB-let-

7a to generate the respective plasmids with the 5'UTR of *p27*. siRL (previously described by [2]) or siAGO2, modified as previously described by [3] { Sense 5' r(CCGUCCCAGAU**G**UCA**G**ACUU)dT, Anti-sense 5' r(AAGU**C**UGA**C**AUCUGGGACGG)dT} was used for transfection at 100 nM per well of a 40% confluent 12 well plate. siDicer1 was from Dharmacon.

Polysome Analysis For polysome analysis, approx. 1 x 10^7 RAW 264.7 cells were lysed in a buffer containing 10 mM HEPES pH 8.0, 25 mM KCl, 5 mM $MgCl_2$, 1 mM DTT, 5mM vanadyl ribonucleoside complex, 1% Triton X-100, 1% sodium deoxycholate, and 1X EDTA-free protease inhibitor cocktail (Roche) supplemented with Cycloheximide (100 µg/ml; Calbiochem). Gradient analysis was performed as described previously [2, 7]. The absorbance profile was obtained by using an ISCO UA-6 absorbance monitor and fractions were collected on an ISCO gradient fractionator. RNA and proteins from the individual fractions were isolated and analyzed following previously published procedure [7]. For isolation of total polysomal pool, pre-cleared RAW 264.7 cell lysate was ultracentrifuged on a 30% sucrose cushion at 100000 Xg using a SW-60 Ti rotor for 1 h. The non-polysomal supernatant was collected from top of the cushion. The sucrose cushion was diluted with dilution buffer (10 mM HEPES pH 8.0, 25 mM KCl, 5 mM $MgCl_2$, 1 mM DTT, 5mM vanadyl ribonucleoside complex, and 1X EDTA-free protease inhibitor cocktail), ultracentrifuged for 30 min and the polysomal pellet was finally dissolved in polysome buffer for further experiments.

Immunofluorescence For immunofluorescence analysis, cells were fixed with 4% paraformaldehyde for 30 min, permeabilized and blocked with PBS containing 1% BSA and 0.1% Triton X-100 and 10% goat sera (GIBCO) for 30 min. The anti-Ago2, anti-Dcp1a and anti-HA antibody were used at 1:100 dilutions. The anti-RCK/p54 and anti-gp63 antibody were used at 1:1000 dilution. Secondary anti-rabbit, anti-mouse or anti-rat antibodies labeled either with Alexa Fluor® 488 dye (green), Alexa Fluor® 594 dye (red) fluorochromes

(Molecular Probes) were used at 1:500 dilutions. Cells were observed under a Plan Apo VC 60X/1.40 oil or Plan Fluor 10X/0.30 objectives on an inverted Eclipse Ti Nikon microscope equipped with a Nikon Qi1MC or QImaging-Rolera EMC^2 camera for image capture.

ChIP Assay ChIP assay was done as described previously [8] . Shortly, 1% formaldehyde was added directly to the medium for 15 min to cross-link nuclear proteins to DNA. Glycine was added to final concentration 0.125 M and incubated for 5 min to avoid over-crosslinking. After PBS wash, cells were collected in ice-cold PBS supplemented with a protease inhibitor cocktail. Cells were lysed with lysis buffer (1% SDS, 10 mM EDTA, protease inhibitors and 50 mM Tris–HCl, pH 8.1) and the lysates were sonicated to obtain DNA fragments of 300–1000 bp in length. Cellular debris was removed by centrifugation and the lysates were diluted 1:10 in ChIP dilution buffer (0.01% SDS, 1.1% Triton X-100, 1.2 mM EDTA, 167 mM NaCl, protease inhibitors and 16.7 mM Tris–HCl, pH 8.1). Non-specific background was removed by incubating the chromatin suspension with a salmon sperm DNA/protein G agarose (Invitrogen) for 30 min at 4°C with agitation. The samples were centrifuged and the recovered chromatin solutions were incubated with indicated antibodies overnight at 4°C with rotation. The antibody against RNA Polymerase II was from Santa Cruz Biotechnology and HA antibody was from Roche, Germany. The immuno-complexes were collected with 60 μl of protein G agarose (Invitrogen) for 2 h at 4°C with rotation. The beads were washed sequentially for 5 min by rotation with 1 ml of the following buffers: low-salt wash buffer (0.1% SDS, 1% Triton X-100, 2 mM EDTA, 150 mM NaCl and 20 mM Tris–HCl, pH 8.1), high-salt wash buffer (0.1% SDS, 1% Triton X-100, 2 mM EDTA, 500 mM NaCl and 20 mM Tris–HCl, pH 8.1) and LiCl wash buffer (0.25 mM LiCl, 1% Triton X-100, 1% sodium deoxycholate, 1 mM EDTA and 10 mM Tris–HCl, pH 8.1). Finally, the beads were washed twice with 1 ml TE buffer (1 mM EDTA and 10 mM Tris–HCl, pH 8.0). The cross-linking was reversed by adding Chelex-100 and incubated for 10 min at 100°C. The remaining proteins were digested by adding proteinase K (final concentration 40 μg/ml) and incubated for 30 min at 55°C. The DNA was recovered by phenol/chloroform/isoamyl alcohol (25:24:1)

extractions and precipitated with 0.1 vol of 3 M sodium acetate, pH 5.2 and 2 vol of ethanol using glycogen as a carrier.

Nuclear Fractionation To isolate nuclei, cells were treated with 10 μM Cytochalasin B for 10 min at 37° C. Cells were washed with ice-cold PBS and lysed in lysis buffer (10 mM HEPES, pH 7.4, 10 mM KCL, 2 mM $MgCl_{2,}$ 1 mM DTT, 10 μM Cytochalasin B, protease Inhibitor cocktail, 0.5% Triton X-100) at 4 ^{o} C for 20 min. Cell lysate was layered over 30% sucrose in Nuclei Buffer (10 mM HEPES, pH 7.4, 10 mM KCL, 2 mM $MgCl_{2,}$ 1 mM DTT, 10 μM Cytochalasin B, protease Inhibitor cocktail) and centrifuged at 1000X g for 20 min. The post-nuclear sup was collected and the centrifugation step was repeated. The pellet was dissolved in Nuclei Buffer and used for western Blotting. The antibody against HuR was from SantaCruz Biotechnology.

Optiprep Gradient Analysis Optiprep^{TM} (Sigma-Aldrich, USA) was used to prepare a 3-30% continuous gradient in a buffer containing 78 mM KCl,4 mM $MgCl_2$,8.4 mM $CaCl_2$,10 mM EGTA, 50 mM Hepes (pH 7.0) for separation of subcellular organelles as described previously [9] with minor modifications described below. Cells were washed with PBS and homogenized with a Dounce homogenizer in a buffer containing 0.25 M sucrose,78 mM KCl,4 mM $MgCl_2$,8.4 mM $CaCl_2$,10 mM EGTA,50 mM Hepes pH 7.0 supplemented with 100 μg/ml of Cycloheximide 5 mM Vanadyl Ribonucleoside Complex (VRC) (Sigma Aldrich), 0.5 mM DTT and 1X Protease Inhibitor. The lysate was clarified by centrifugation at 1000g for 5 minutes two times and layered on top of the prepared gradient. The tubes were centrifuged at 36,000 rpm in SW60 rotor for 5 hr for separation of gradient and ten fractions were collected by aspiration from the top for subsequent analysis of proteins and RNA.

Computational Modeling The three dimensional coordinates for human hAgo1 (NP_036331) and hAgo3 (NP_079128) proteins were generated using the hAgo2 structure (PDB ID: 4FT3) [10] as reference via a homology modeling software named MODELLER v 9.8 [11]. Probable binding cavities of Argonaute proteins were identified using the CASTp

[12] program. The ATP coordinates bound with mid domain of hAgo2 protein (PDB ID: 3QX9) [13] were extracted and superimposed with the full length hAgo2 structure (PDB ID: 4F3T) [10] to generate the complete hAgo2 and ATP complex. Similar way hAgo3 and ATP complex was also generated. The ATP bound full hAgo2 and hAgo3 structures were then subjected to a two-step energy minimization procedure (steepest descent followed by conjugate gradient protocol) in order to relieve any unfavorable structural clashes between the Argonaute proteins and bound ATP molecule. GROMACS molecular simulation package was [14] employed using The GROMOS53a6 force-field to define the basic structural properties of the protein and ATP while solvated within water. Once the stable ATP bound structures of hAgo2 and hAgo3 were obtained, a ligand (ATP) binding/unbinding pathway study for both proteins were performed using the MoMA-Ligpath program [15]. MoMA-Ligpath enables the simulation of ligand binding/unbinding from the active site of a protein within a permissible computational time. Each binding/unbinding step of ATP from the binding sites of hAgo2 and hAgo3 was then used to estimate the interaction energy following a semi empirical quantum chemistry package, MOPAC [16]. In each step of binding/unbinding, a distance cut-off of 3.5 Å from the ATP was used to find the potential interacting protein atoms with the ATP. With this defined protein-ATP interaction, binding/unbinding energies in term of the thermodynamic properties were calculated using the MOZYME method and PM7 semi empirical Hamiltonian parameters [16] embedded within MOPAC software. All the structural figure and movie generation were done using the chimera [17] and PyMOL software (http://pymol.org/).

Post imaging analysis All western blot and Northern blot images were processed with Adobe Photoshop CS4 for all linear adjustments and cropping. All images captured on Nikon Eclipse Ti microscope and processed with Nikon NIS ELEMENT AR 3.1 software. Cropping was done using Adobe Photoshop CS4.

Table S1: List of Primers used

Target	Forward Primer	Reverse Primer
TNF-α	5’- GTCTCAGCCTCTTCTCATTCC -3’	5’-TCCACTTGGTGGTTTGCTACG -3’
IL-1□	5’-GACCTTCCAGGATGAGGACAT-3’	5’-CCTTGTACAAAGCTCATGGAG–3’
IL-6	5’- AGGATACCACTCCCAACAGA -3’	5’- GTACTCCAGAAGACCAGAGGA-3’
IL-10	5’-TGCTAACCGACTCCTTAATGC-3’	5’-ATCACTCTTCACCTGCTCCAC-3’
TLR4	5’-GGAGAACAAAACTCTGGGGC-3’	5'-TGGCTTGTATTCAAAAGGCCCAGT-3'
RL Reporters	5' CCAAGCAAGATCATGC 3'	5' GCTCTTGATGTACTTACCC 3'
mAgo1	5'TGT GAG CTG GCG CAT GCT GC 3'	5'- GTA GTA GCC CTC AGG CGG TG -3'
mAgo2	5'- AGG CTG TTC CAA CCC TCT GG -3'	5'- CCT TTG GGA ATC TGT CAG AGG -3'
mAgo3	5'- GTG TCT CGA GTC AGC TGG CAC -3'	5'- GTG GTC ATA TCC TTC TGG AGC -3'
mAgo4	5'- CAA AGT GTC TGT GCA GTG G -3'	5'- CCC AGA GGG TGG TAA TAA CC -3'
18S rRNA	5' TGACTCTAGATAACCTCGGG 3'	5' GACTCATTCCAATTACAGGG 3'

Table S2: List of Antibodies used

Name of Antigen	Raised in	Source	Dilutions
4G10 (Anti-phosphotyrosine)	Mouse	Millipore	1:1000
4A4 (Anti-phosphoserine)	Mouse	Millipore	1:1000
GW182	Rabbit	Bethyl	1:2000
GFP	Mouse	Roche	1:1000
β-Actin	Mouse	Sigma	1:10000
HA	Rat Monoclonal	Roche	1:1000
AGO2 (eIF2C2)	Mouse	Novus Biologicals	1:500
RCK/p54	Rabbit	Bethyl	1:5000
Dcp1a	Mouse	Novus Biologicals	1:1000
HuR	Mouse	SantaCruz	1:1000
LAMP1	Rabbit	Cell Signalling	1:1000
HRS	Rabbit	Bethyl	1:1000

Supplementary figure legends

Figure S1| Derepression of miRNA activity in mammalian macrophage cells.

(A) Fold repression of let-7a reporters. RL3xbulge-let7a-Mut and RL HMGA2 3'UTR-Mut reporters with mutated let-7a binding sites were used to calculate fold repression value (*left panel*). Fold repression of RLHMGA2 3'UTR reporter in the presence of let-7a mimic (*right panel*). (**B**) Repression level change of a GFP reporter with miRNA let-7a sites in RAW 264.7 cells upon LPS treatment. Control and reporter plasmids were transfected to RAW 264.7 cells and stimulated with LPS for indicated duration before they were extracted and western blotted for GFP. β-Actin served as loading control. (**C**) Alteration of miRISC upon LPS treatment *in vivo*. RAW 264.7 cells were transfected either with RL-per-let-7a having one let-7a perfect binding site or with RL-con reporter and fold repression values were calculated at each time point. In a separate set of experiments RL-con reporter was transfected either with siRL, a siRNA targeting coding sequence of RL mRNA, or with control siRNA and luciferase assays were done after LPS treatment. (**D**) Repression of RL3xbulge-miR-122 in THP-1 cells expressing miR-122 before and after LPS treatment. (**E**) Deactivation of miRNP with other proinflammatory agents. RAW 264.7 cells were stimulated with either CpG or PMA and level of repression was measured before and after the treatment. (**F**) LPS isolated from two different sources had similar effect on let-7a mediated repression. RAW 264.7 cells were stimulated either with *E. coli* LPS (1ng/ml) or *Salmonella typhimurium* LPS (1ng/ml) for different durations and luciferase assays were done. (**G**) Effect of Dicer1 knockdown on TNF-α expression in naïve and LPS stimulated RAW 264.7 cells.

Figure S2| LPS stimulation does not change the levels of let-7a or other miRNP components but alters subcellular localization of Ago2.

(**A**) Expression of let-7a or exogenously expressed miR-122 in RAW 264.7 cells subjected to LPS treatment. Northern blots were done to detect let-7a or exogenous miR-122 in naïve and LPS treated RAW 264.7 cells. U6 snRNA served as loading control. let-7a miRNA levels in primary mouse macrophage cells before and after LPS stimulation. Let-7a miRNA level

was also measured quantitatively by Real-Time PCR in RAW 264.7 cells treated with LPS from two different sources. (**B**) miRNP and P-body components expression during LPS treatment. Western blot analysis for Ago2 and two other P-body components Dcp1a and GW182 were done with extracts of LPS treated RAW 264.7 cells. β-Actin served as loading control (*Upper panel*). No change in Ago2 protein level also in primary mouse macrophage cells upon LPS stimulation (*Lower panel*). (**C**) Relative expression of different mammalian Argonautes in RAW 264.7 cells. Real-time quantification was done to estimate Ago1, 2, 3 and 4 mRNA expressions. (**D**) Localization of Ago2 in naïve and LPS activated RAW 264.7 was determined by indirect immunofluorescence using an Ago2 specific antibody. Cells were also co-stained for RCK/p54 to mark the P-bodies. DAPI was used to stain the nucleus. (**E**) Number of P-bodies showing colocalization for two marker proteins Dcp1a and RCK/p54 were determined by indirect immunoflurescenece in RAW 264.7 cells upon LPS stimulation. (**F**) P-bodies showing Ago2 and RCK/p54 co-localization were counted and plotted for naïve and LPS activated RAW 264.7 cells. (**G**) Subcellular fractionation of naïve and LPS stimulated RAW 264.7 cells. Different subcellular fractions were western blotted for Ago2, GAPDH and HuR (*left panel*). ChIP assay was done to measure association of Ago2 with the promoter element of TNF-alpha gene in RAW 264.7 cells. ChIP with RNA PolII specific antibody was used as a positive control. (**H**) Optiprep density gradient analysis of cell homogenates were done to measure any change in subcellular localization of miRNA in LPS activated RAW 264.7 cells expressing miR-122. LAMP1 was used as lysosomal marker and HRS was used as early endosomal marker. miR-122 was detected by Northern blotting. Percentiles of total amount of miR-122 present in each fraction were determined by densitometric analysis and were plotted both for naïve and LPS treated macrophages.

Figure S3| Active protein translation but not the *de novo* transcription of target mRNA has a role in the derepression of miRNA activity in LPS stimulated macrophages.

(**A**) Level of control and reporter mRNAs was unaltered in LPS treated cells. Real-time quantification of the RL-con and the let-7a reporter was done both in treated and untreated

cells. (**B**) Effect of transcription blockage on derepression of miRNA-targeted messages in LPS treated cells. RAW 264.7 cells were pre-treated with 10mM α-amanitin for 2h followed by LPS stimulation for 4h before the repression level of let-7a reporter was measured (** $P < 0.0019$) (*left panel*). Real-time quantification of the mRNA level of these reporter mRNAs in α-amanitin treated and untreated cells was also done (*right panel*). (**C-D**) Derepression of a let-7a reporter mRNA in RAW 264.7 cells treated with LPS. RL-3xbulge-miR-122 reporter or control mRNA were transfected in miR-122 expressing RAW 264.7 cells followed by LPS stimulation before repression level (**C,** *left panel*) and reporter mRNA levels were measured in naïve and LPS activated cells (**C,** *right panel*). Association of transfected reporter mRNA with Ago2 in naïve and LPS treated cells were also determined (**D**). Normalization of expression level was done against amount of respective mRNAs for calculation of fold repression. (**E**) Polysome profile of let-7a miRNA, its target RL-3xbulge-let-7a and Ago2 protein in naïve and LPS stimulated RAW 264.7 cells. 18S rRNA used as control while a continuous absorption during the fraction collection at 254nm was also plotted. The level of RL or 18S rRNA level in each fraction was measured by a semi-quantitative RT-PCR and let-7a miRNA levels quantified by northern blotting. Ago2 in each fraction was western blotted. (**F**) Effects of Rapamycin on LPS induced derepression of miRNA activity in RAW 264.7 cells. Repression of RL reporter was measured in naïve and LPS stimulated RAW 264.7 cells pre-treated with 50 ng/ml Rapamycin for overnight (*** $P < 0.0001$). (**G**) Slow translation of reporter mRNA affects the efficacy of derepression. The schematic representation of the let-7a RL reporter with the 5'UTR of human *p27* mRNA with a predicted secondary structure that slow down translation from *cis*-encoded mRNA. Fold repression was measured in naïve and LPS stimulated cells (** $P < 0.0009$).

Figure S4| Higher phosphorylation at Y529 and altered GW182 and Dcp1a interaction of Ago2 in LPS activated macrophages.

(**A**) RAW 264.7 cells were transfected with HA-Ago2 construct and let-7a level in HA-Ago2 IPed materials was determined (*upper panel*). let-7a level was also determined from Ago2

IPed materials in THP1 cells (*lower panel*). (**B**) HA-Ago2 association of different miRNAs in RAW 264.7 cells expressing miR-122. Real-Time quantification from input was done to check miR-122 expression in naïve and LPS treated cells. (**C**) RAW 264.7 cells either transfected with HA-Ago2 or with HA-GW182, were treated with LPS for 4h and IPed with anti-HA antibody. The IPed materials were western blotted to detect the HA-tagged protein and co-IPed GW182 or Ago2 were detected with antibodies specific for these proteins. (**D**) RAW 264.7 cells were transfected with HA-Ago2, IPed with anti-HA antibody and western blotted for phosphorylated Ago2 with 4G10 antibody (*top panel*). LPS stimulated primary macrophage cell extracts were IPed with anti-Ago2 antibody and western blotted for phosphorylated Ago2, GW182 and Dcp1a (*bottom panel*). (**E**) Factors present in LPS treated RAW 264.7 cell extract affects miRNP level *in vitro*. miR-122 miRNP isolated from HEK293 cells expressing FLAG-HA-Ago2 using anti-FLAG antibody coupled beads and were incubated with LPS treated or naïve RAW 264.7 cell extract before the miRNPs were separated, washed and FLAG-HA-Ago2 bound miRNA level was determined. (**F**) The LPS mediated derepression process requires activation of p38 MAPK. Let-7a miRNA level and Tyr phosphorylated HA-Ago2 level were measured in LPS treated RAW 264.7 cells pre-treated with 10μM SB203580, the p38MAPK inhibitor for 2h (* $P < 0.0270$) (*left panel*). Repression of RL reporter was measured in naïve and LPS stimulated RAW 264.7 cells pre-treated with 10μM SB203580 or DMSO for 2 h (* $P < 0.0193$) (*right top panel*). Similar experiments were done in presence of ERK or JNK specific inhibitors (*right bottom panel*). (**G**) Level of Tyr phosphorylation of HA-Ago3 expressed in naïve and LPS activated RAW 264.7 cells (*Upper panel*). The amount of miRNA target mRNA bound to HA-Ago3 IPed from naïve and LPS treated macrophage (*bottom panel*). **(H)** LPS stimulated RAW 264.7 cell extract were used to IPed Ago2 either with anti-Ago2 or anti-HA antibodies (for FHA-Ago2 or mutant expressing cells) and western blotted for Serine phosphorylated Ago2 with anti-phosphoserine specific antibody. In all immunoprecipitation experiments quantifications were perfomed by densitometric estimation of bands normalized against signals obtained for heavy chain of IgG used for immunoprecipitation.

Figure S5| hAgo2 and hAgo3 proteins have differences in cavity size and ATP binding affinity.

(**A**) Structural superimposition of 3D models of hAgo1 (NP_036331) and hAgo3 (NP_079128) with the hAgo2 crystal structure (PDB ID: 4FT3). (**B**) Differences in solvent accessible volume and molecular surface volume of the largest cavity identified by the CASTp program [14] for the Argonaute proteins. (**C**) The theoretical binding/unbinding ΔG (Kcal/mol) values of ATP from MID domains of hAgo2 and hAgo3 proteins. The horizontal axis displays the stages (Final, Last 10 Average and All Average) at which the ΔG values were calculated. "Final" indicates ΔG value of the ATP bound end structure of hAgo2 and hAgo3 proteins; "Last 10 Average" shows the average ΔG value of ATP with the respective proteins, calculated from the last 9 steps of ATP binding path and ultimately merging with the end structure (a total 9+1 steps). The "All Average" shows the average ΔG value of ATP obtained from all the steps of binding. In every case hAgo2 shows a lower ΔG value as compared to hAgo3 indicating a better interaction with ATP. The simulated ATP binding/unbinding steps were obtained from MoMA-Ligpath [15] and ΔG values were calculated by using MOPAC semiempirical quantum chemical package [16]. (**D**) Percentage of amino acids surrounding the ATP binding/unbinding path. The figure shows a notable difference in amino acid types surrounding the ATP binding/unbinding path of hAgo2 and hAgo3 proteins, a possible explanation for the observed difference in ΔG values in Fig S5C. (**E**, **F** and **G**) Relative position of phosphorylated Serine 385, 387 and 388 of hAgo1, hAgo2 and hAgo3 respectively in cyan with Tyrosine 527, 529 and 530 of hAgo1, hAgo2 and hAgo3 are shown in blue. In all the cases the measured distance between aforementioned Serine and Tyrosine residue is found to be 49 Å. (**H**) Optiprep density gradient analysis was done to identify any change in sub-organelle localization of HA-Ago2 and HA-Ago3 protein in LPS activated macrophages. HRS was used as marker of endosomes.

Supplementary References

1. Kundu P, Fabian MR, Sonenberg N, Bhattacharyya SN, Filipowicz W (2012) HuR protein attenuates miRNA-mediated repression by promoting miRISC dissociation from the target RNA. *Nucleic Acids Res* **40:** 5088-5100

2. Pillai RS, Bhattacharyya SN, Artus CG, Zoller T, Cougot N, Basyuk E, Bertrand E, Filipowicz W (2005) Inhibition of translational initiation by Let-7 MicroRNA in human cells. *Science* **309:** 1573-1576

3. Rudel S, Wang Y, Lenobel R, Korner R, Hsiao HH, Urlaub H, Patel D, Meister G (2011) Phosphorylation of human Argonaute proteins affects small RNA binding. *Nucleic Acids Res* **39:** 2330-2343

4. Meister G, Landthaler M, Patkaniowska A, Dorsett Y, Teng G, Tuschl T (2004) Human Argonaute2 mediates RNA cleavage targeted by miRNAs and siRNAs. *Mol Cell* **15:** 185-197

5. Chang J *et al* (2004) miR-122, a mammalian liver-specific microRNA, is processed from hcr mRNA and may downregulate the high affinity cationic amino acid transporter CAT-1. *RNA Biol* **1:** 106-113

6. Lee YS, Dutta A (2007) The tumor suppressor microRNA let-7 represses the HMGA2 oncogene. *Genes Dev* **21:** 1025-1030

7. Bhattacharyya SN, Habermacher R, Martine U, Closs EI, Filipowicz W (2006) Relief of microRNA-mediated translational repression in human cells subjected to stress. *Cell* **125:** 1111-1124

8. Sinkkonen L, Malinen M, Saavalainen K, Vaisanen S, Carlberg C (2005) Regulation of the human cyclin C gene via multiple vitamin D3-responsive regions in its promoter. *Nucleic Acids Res* **33:** 2440-2451

9. Gibbings DJ, Ciaudo C, Erhardt M, Voinnet O (2009) Multivesicular bodies associate with components of miRNA effector complexes and modulate miRNA activity. *Nat Cell Biol* **11:** 1143-1149

10. Elkayam E, Kuhn CD, Tocilj A, Haase AD, Greene EM, Hannon GJ, Joshua-Tor L (2012) The structure of human argonaute-2 in complex with miR-20a. *Cell* **150:** 100-110

11. Eswar N, Webb B, Marti-Renom MA, Madhusudhan MS, Eramian D, Shen MY, Pieper U, Sali A (2007) Comparative protein structure modeling using MODELLER. *Curr Protoc Protein Sci* **Chapter 2:** Unit 2 9

12. Dundas J, Ouyang Z, Tseng J, Binkowski A, Turpaz Y, Liang J (2006) CASTp: computed atlas of surface topography of proteins with structural and topographical mapping of functionally annotated residues. *Nucleic Acids Res* **34:** W116-118

13. Frank F, Fabian MR, Stepinski J, Jemielity J, Darzynkiewicz E, Sonenberg N, Nagar B (2011) Structural analysis of 5'-mRNA-cap interactions with the human AGO2 MID domain. *EMBO Rep* **12:** 415-420

14. Pronk S *et al* (2013) GROMACS 4.5: a high-throughput and highly parallel open source molecular simulation toolkit. *Bioinformatics* **29:** 845-854

15. Devaurs D, Bouard L, Vaisset M, Zanon C, Al-Bluwi I, Iehl R, Simeon T, Cortes J (2013) MoMA-LigPath: a web server to simulate protein-ligand unbinding. *Nucleic Acids Res* **41:** W297-302

16. Stewart JJ (2013) Optimization of parameters for semiempirical methods VI: more modifications to the NDDO approximations and re-optimization of parameters. *J Mol Model* **19:** 1-32

17. Pettersen EF, Goddard TD, Huang CC, Couch GS, Greenblatt DM, Meng EC, Ferrin TE (2004) UCSF Chimera--a visualization system for exploratory research and analysis. *J Comput Chem* **25:** 1605-1612

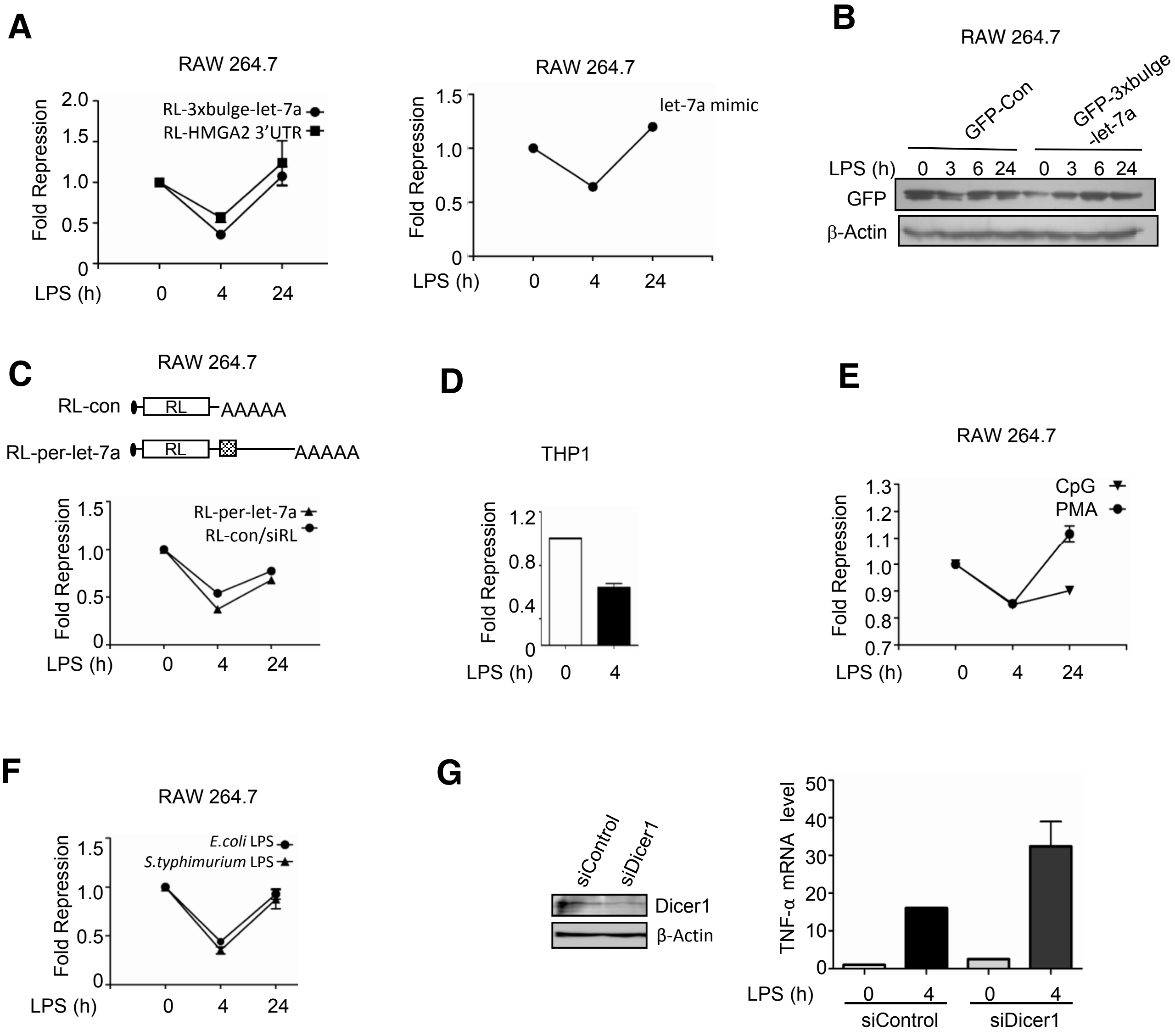
A
RAW 264.7
RL-3xbulge-let-7a
RL-HMGA2 3'UTR
Fold Repression
LPS (h)
RAW 264.7
let-7a mimic
B
RAW 264.7
GFP-Con
GFP-3xbulge -let-7a
LPS (h) 0 3 6 24 0 3 6 24
GFP
β-Actin
C
RAW 264.7
RL-con
RL-per-let-7a
RL
AAAAA
RL-con/siRL
D
THP1
E
RAW 264.7
CpG
PMA
F
RAW 264.7
E.coli LPS
S.typhimurium LPS
G
siControl
siDicer1
Dicer1
β-Actin
TNF-α mRNA level
siControl
siDicer1

Figure S1

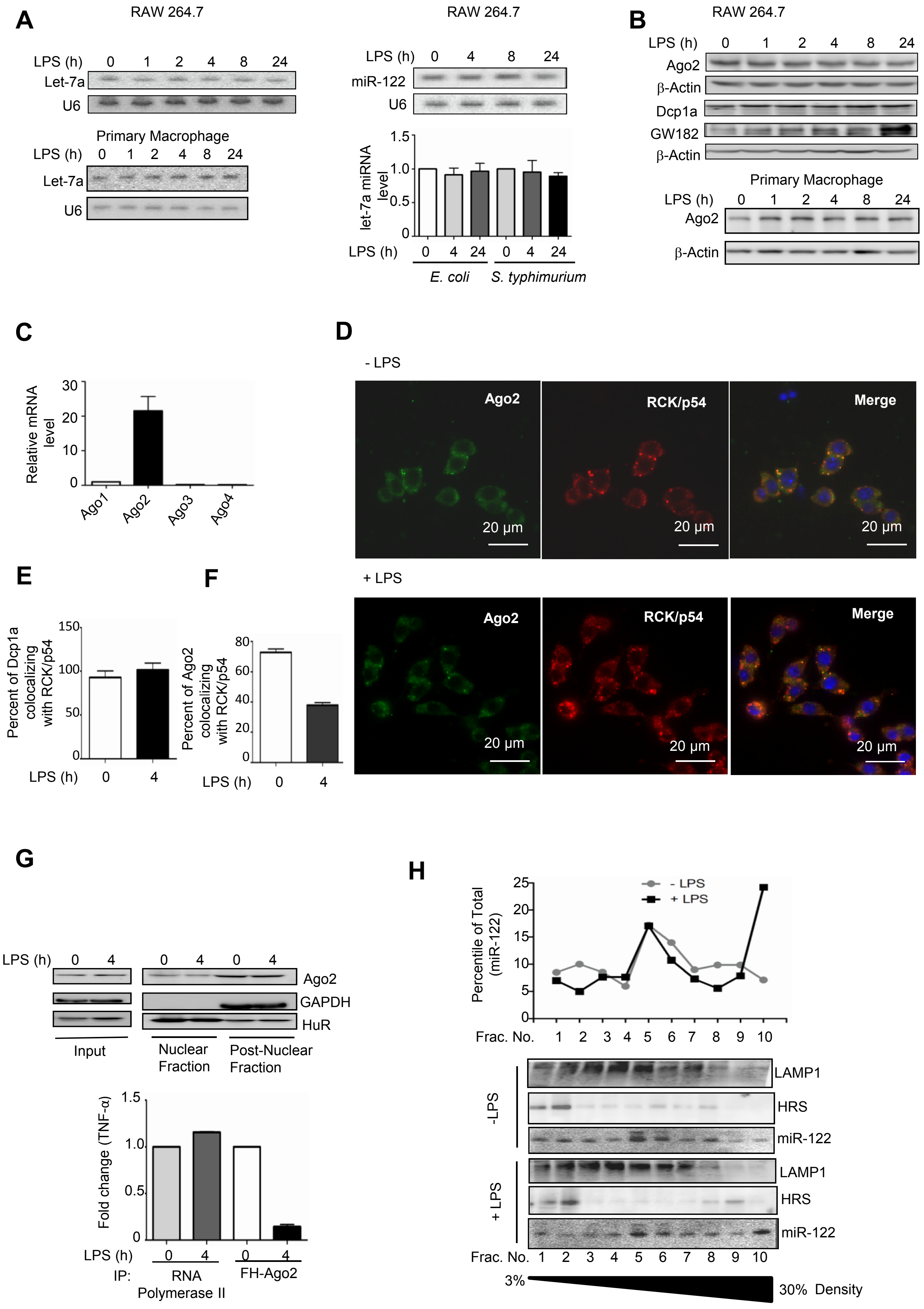
A
RAW 264.7
LPS (h) 0 1 2 4 8 24
Let-7a
U6
Primary Macrophage
RAW 264.7
LPS (h) 0 4 8 24
miR-122
let-7a miRNA level
E. coli
S. typhimurium
B
Ago2
β-Actin
Dcp1a
GW182
C
Relative mRNA level
Ago1
Ago2
Ago3
Ago4
D
- LPS
+ LPS
RCK/p54
Merge
20 μm
E
Percent of Dcp1a colocalizing with RCK/p54
F
Percent of Ago2 colocalizing with RCK/p54
G
GAPDH
HuR
Input
Nuclear Fraction
Post-Nuclear Fraction
Fold change (TNF-α)
IP:
RNA Polymerase II
FH-Ago2
H
Percentile of Total (miR-122)
Frac. No.
LAMP1
HRS
-LPS
+ LPS
3%
30% Density

Figure S2

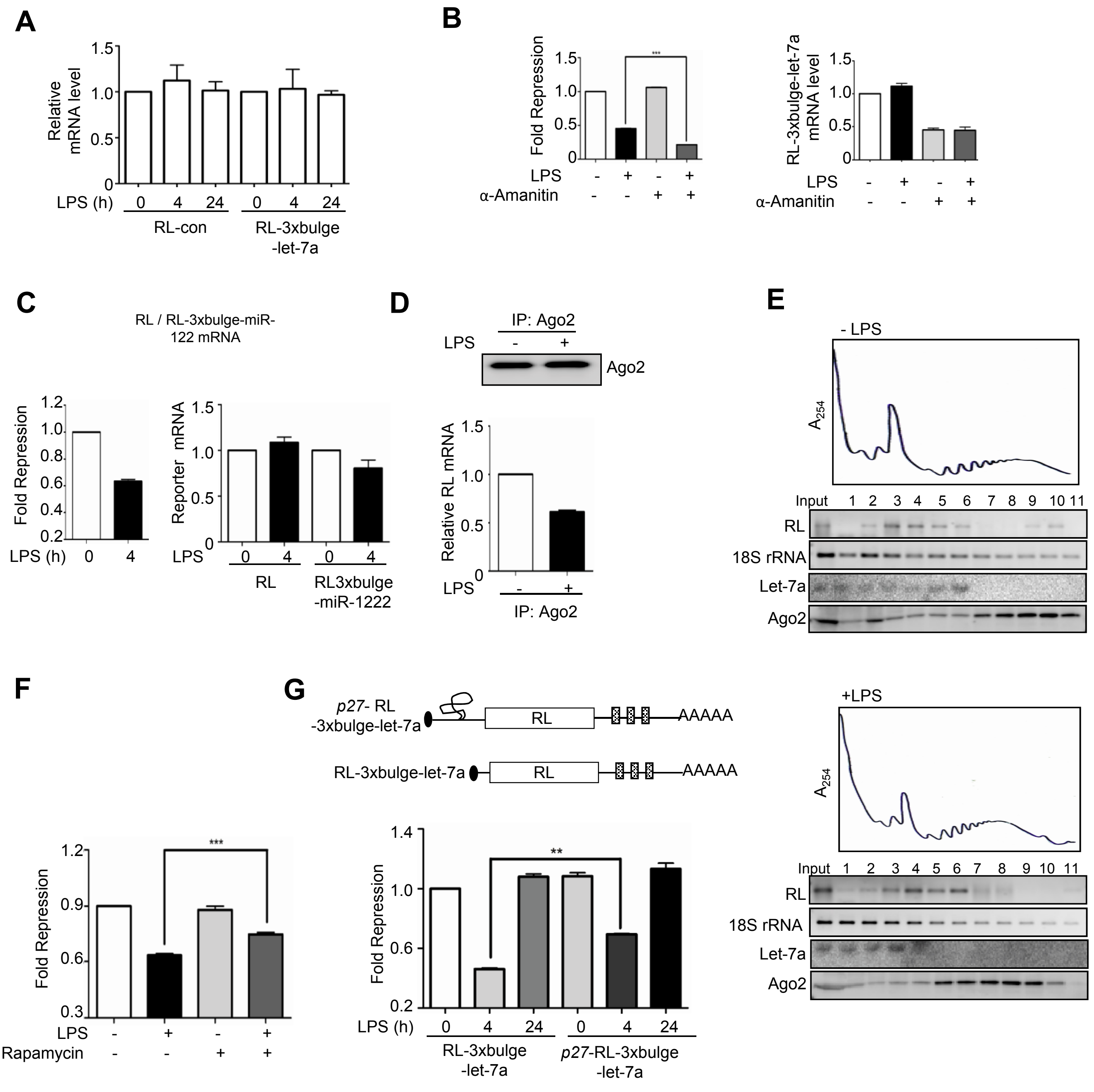

A
Relative mRNA level
LPS (h) 0 4 24 0 4 24
RL-con
RL-3xbulge -let-7a
B
Fold Repression
LPS - + - +
α-Amanitin - - + +
RL-3xbulge-let-7a mRNA level
C
RL / RL-3xbulge-miR-122 mRNA
Fold Repression
LPS (h) 0 4
Reporter mRNA
RL
RL3xbulge -miR-1222
D
IP: Ago2
Ago2
Relative RL mRNA
E
- LPS
+LPS
A254
Input 1 2 3 4 5 6 7 8 9 10 11
RL
18S rRNA
Let-7a
Ago2
F
Fold Repression
LPS
Rapamycin
G
p27- RL -3xbulge-let-7a
RL-3xbulge-let-7a
AAAAA
RL-3xbulge -let-7a
p27-RL-3xbulge -let-7a

Figure S3

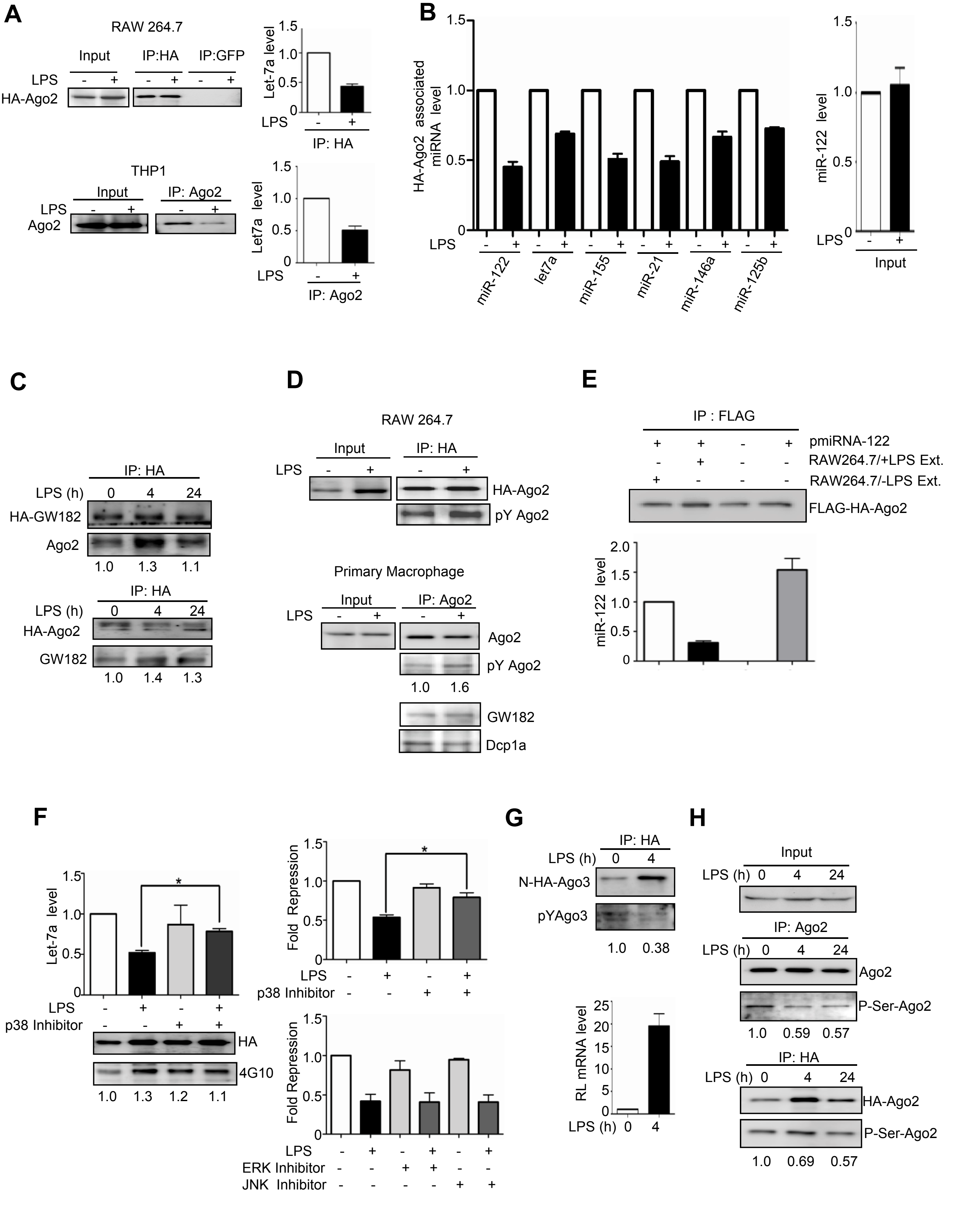
A
RAW 264.7
Input
IP:HA
IP:GFP
LPS
HA-Ago2
Let-7a level
IP: HA
THP1
Input
IP: Ago2
LPS
Ago2
Let7a level
IP: Ago2
B
HA-Ago2 associated miRNA level
LPS
miR-122
let7a
miR-155
miR-21
miR-146a
miR-125b
miR-122 level
Input
C
IP: HA
LPS (h) 0 4 24
HA-GW182
Ago2
1.0 1.3 1.1
IP: HA
LPS (h) 0 4 24
HA-Ago2
GW182
1.0 1.4 1.3
D
RAW 264.7
Input
IP: HA
LPS
HA-Ago2
pY Ago2
Primary Macrophage
Input
IP: Ago2
LPS
Ago2
pY Ago2
1.0 1.6
GW182
Dcp1a
E
IP : FLAG
pmiRNA-122
RAW264.7/+LPS Ext.
RAW264.7/-LPS Ext.
FLAG-HA-Ago2
miR-122 level
F
Let-7a level
LPS
p38 Inhibitor
HA
4G10
1.0 1.3 1.2 1.1
Fold Repression
LPS
p38 Inhibitor
Fold Repression
LPS
ERK Inhibitor
JNK Inhibitor
G
IP: HA
LPS (h) 0 4
N-HA-Ago3
pYAgo3
1.0 0.38
RL mRNA level
LPS (h) 0 4
H
Input
LPS (h) 0 4 24
IP: Ago2
LPS (h) 0 4 24
Ago2
P-Ser-Ago2
1.0 0.59 0.57
IP: HA
LPS (h) 0 4 24
HA-Ago2
P-Ser-Ago2
1.0 0.69 0.57

Figure S4

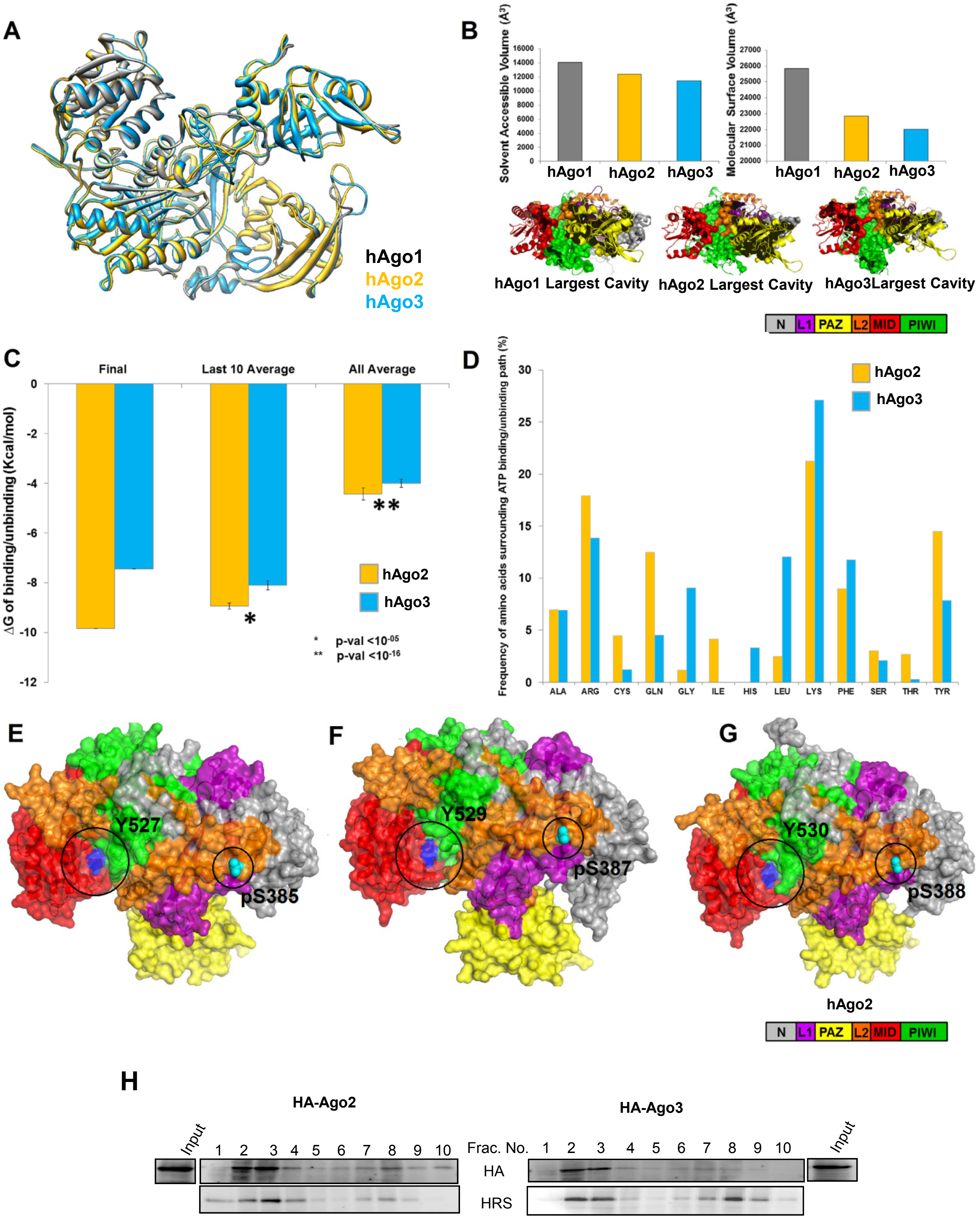
A
hAgo1
hAgo2
hAgo3
B
Solvent Accessible Volume (Å³)
Molecular Surface Volume (Å³)
hAgo1
hAgo2
hAgo3
hAgo1 Largest Cavity
hAgo2 Largest Cavity
hAgo3Largest Cavity
N
L1
PAZ
L2
MID
PIWI
C
Final
Last 10 Average
All Average
ΔG of binding/unbinding (Kcal/mol)
hAgo2
hAgo3
* p-val <10-05
** p-val <10-16
D
Frequency of amino acids surrounding ATP binding/unbinding path (%)
ALA
ARG
CYS
GLN
GLY
ILE
HIS
LEU
LYS
PHE
SER
THR
TYR
E
Y527
pS385
F
Y529
pS387
G
Y530
pS388
hAgo2
H
HA-Ago2
HA-Ago3
Input
Frac. No.
HA
HRS

Figure S5
