# Supplementary material for: A transient reversal of miRNA-mediated repression controls macrophage activation
Source: EMBO Rep. 2013 Sep 13;14(11):1008–16. doi: 10.1038/embor.2013.149 (PMC3851954; doi:10.1038/embor.2013.149)
Supplement: Supplementary Information [file embor2013149dfs2.pdf]

A transient reversal of miRNA-mediated repression controls macrophage activation

Anup Mazumder[a], Mainak Bose[a], Abhijit Chakraborty[b], Saikat Chakrabarti[b] and Suvendra N. Bhattacharyya[a,1]

[a]RNA Biology Research Laboratory, Molecular and Human Genetics Division, CSIR-Indian Institute of Chemical Biology,
Kolkata - 700032, India.
[b]Structural Biology and Bioinformatics Division, CSIR-Indian Institute of Chemical Biology, Kolkata - 700032, India.

[1]To whom correspondence should be addressed

Fig S2A

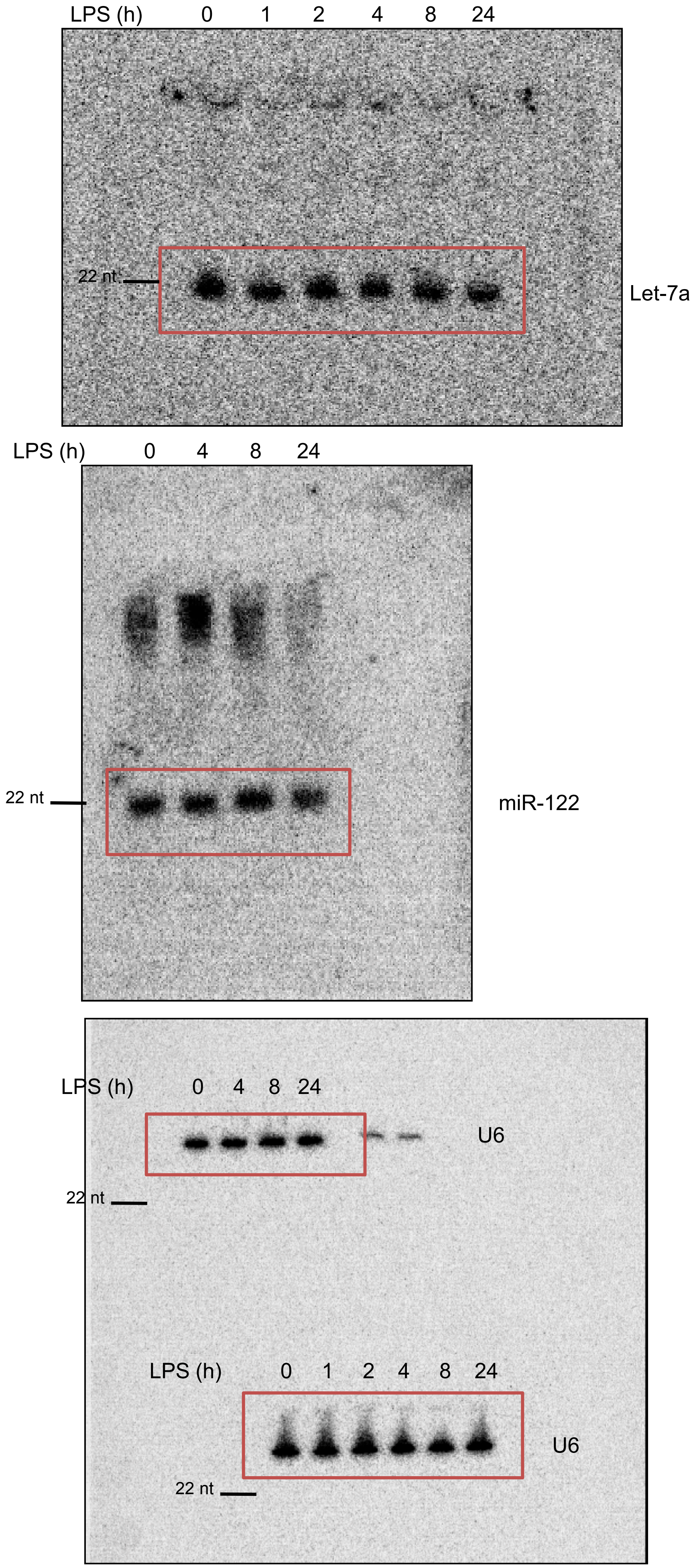

LPS (h) 0 1 2 4 8 24
22 nt
Let-7a
LPS (h) 0 4 8 24
22 nt
miR-122
LPS (h) 0 4 8 24
U6
22 nt
LPS (h) 0 1 2 4 8 24
U6
22 nt

Fig S2B

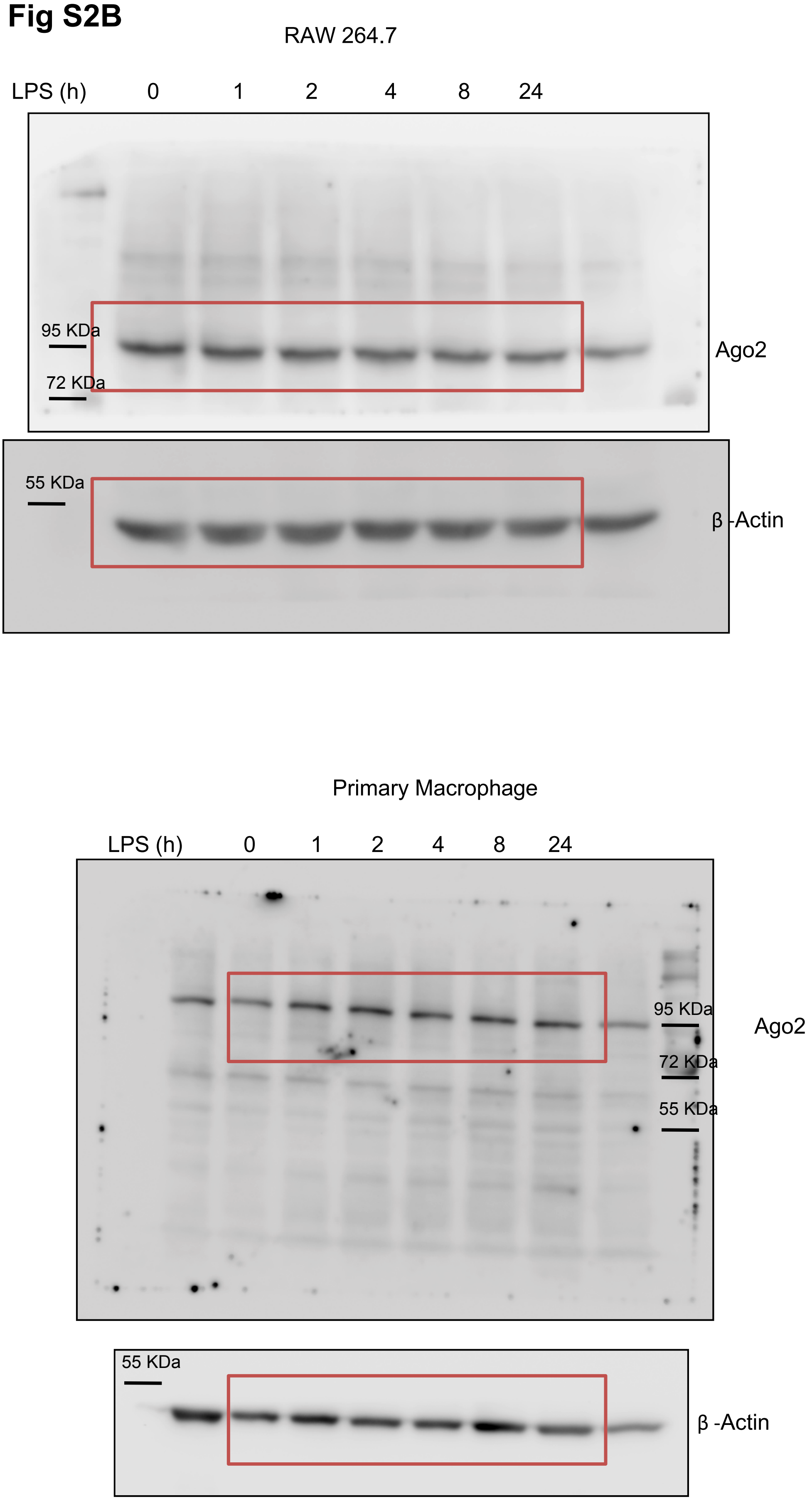
RAW 264.7
LPS (h)
0
1
2
4
8
24
95 KDa
72 KDa
Ago2
55 KDa
β -Actin
Primary Macrophage
LPS (h)
0
1
2
4
8
24
95 KDa
72 KDa
55 KDa
Ago2
55 KDa
β -Actin

Fig S2D

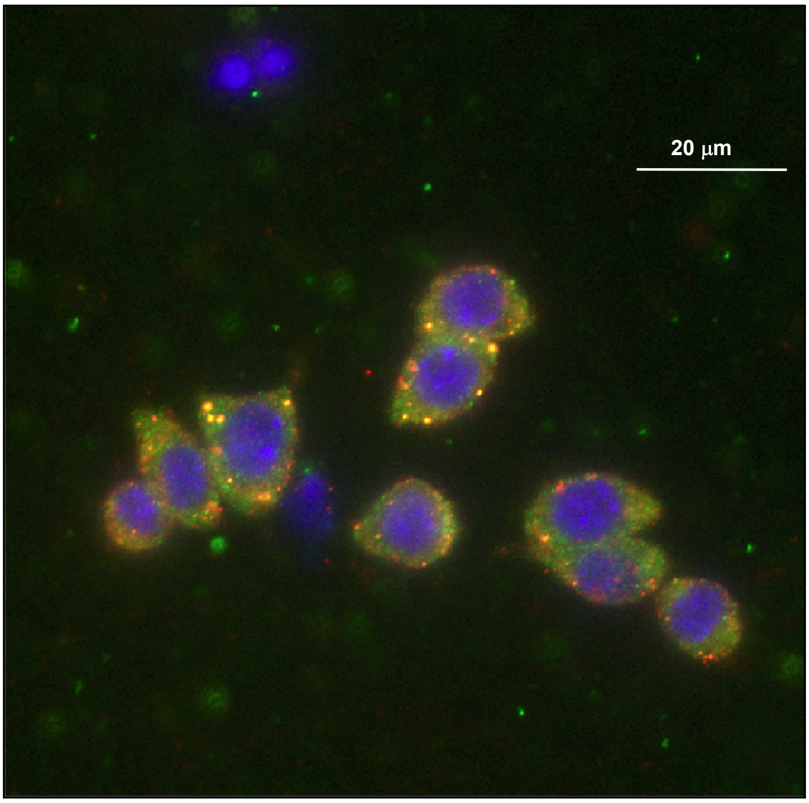

- LPS, 0 hr

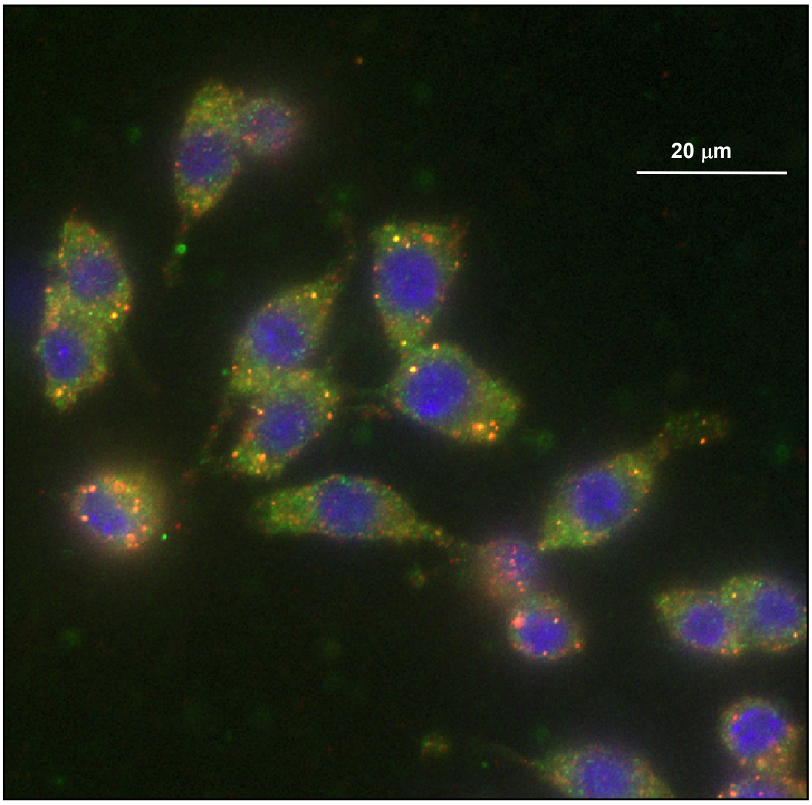

+ LPS, 4 hr

Fig S2H

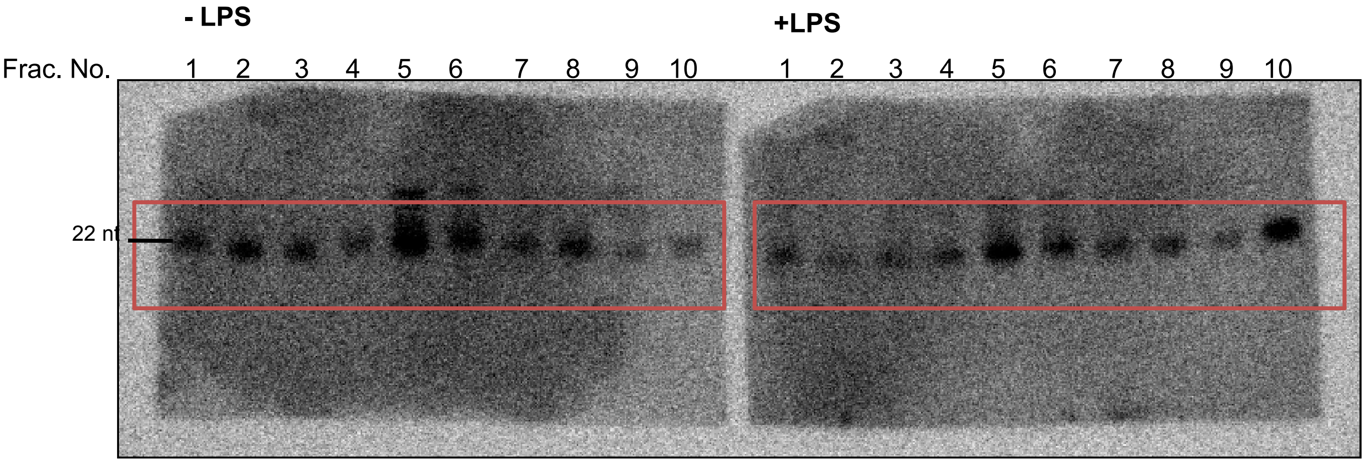

miR-122

Fig S4F

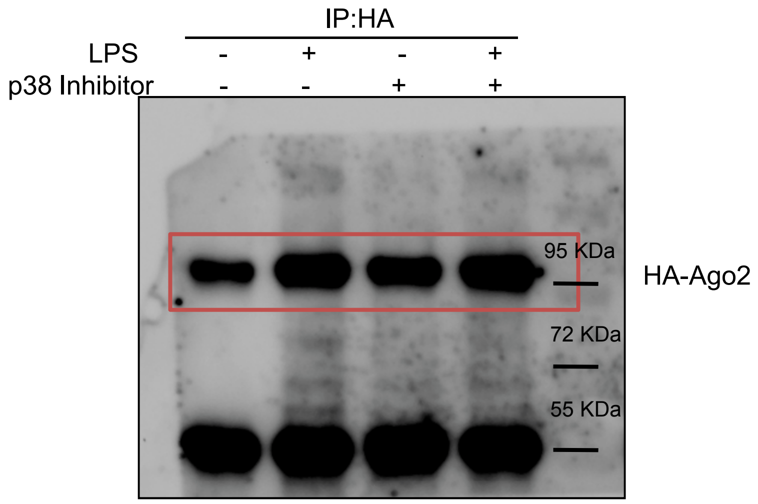
IP:HA
LPS - + - +
p38 Inhibitor - - + +
95 KDa
72 KDa
55 KDa
HA-Ago2

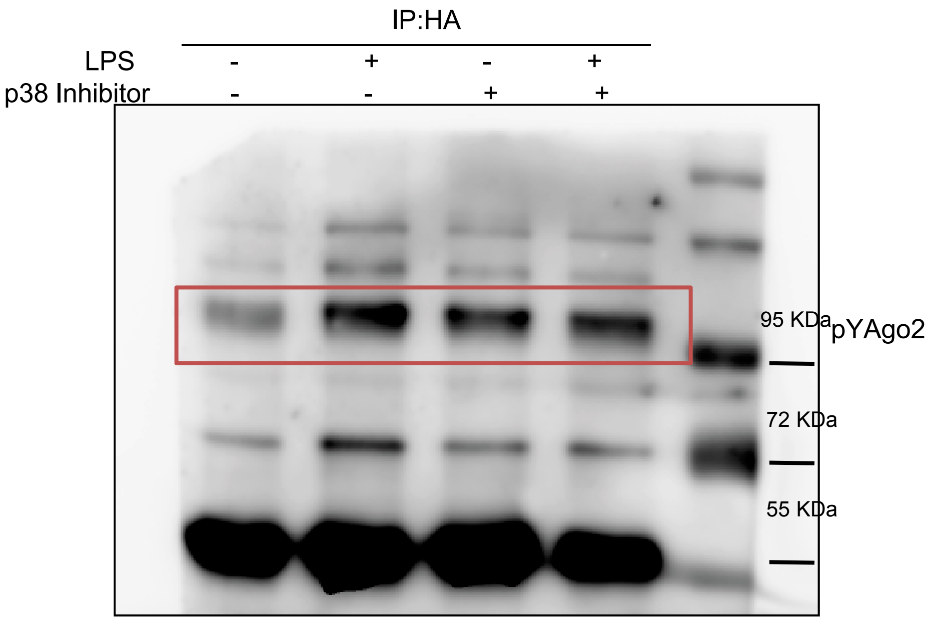
IP:HA
LPS - + - +
p38 Inhibitor - - + +
95 KDa
72 KDa
55 KDa
pYAgo2

Fig S4H

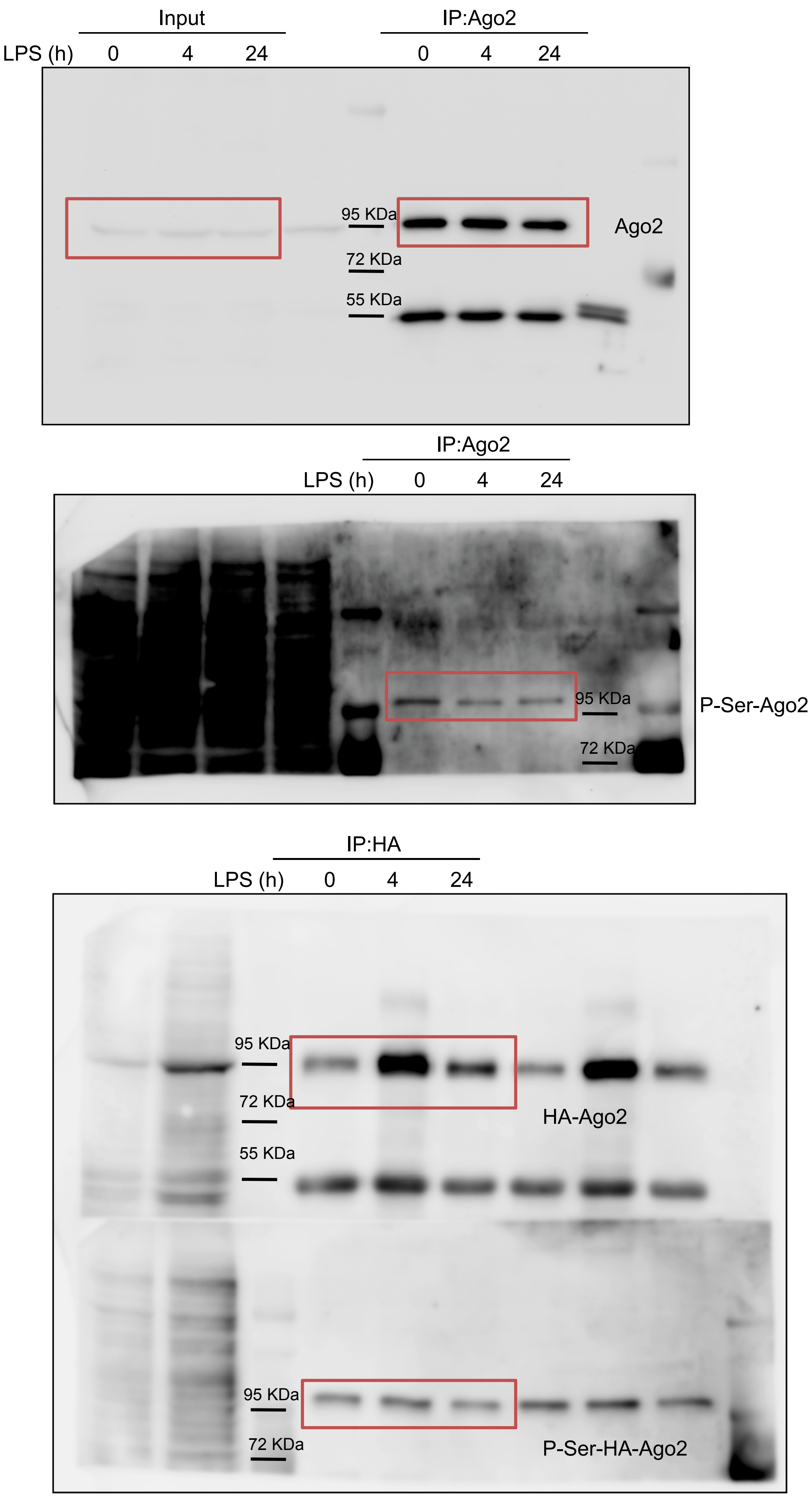
Input
IP:Ago2
LPS (h) 0 4 24
0 4 24
95 KDa
72 KDa
55 KDa
Ago2
IP:Ago2
LPS (h) 0 4 24
95 KDa
72 KDa
P-Ser-Ago2
IP:HA
LPS (h) 0 4 24
95 KDa
72 KDa
55 KDa
HA-Ago2
95 KDa
72 KDa
P-Ser-HA-Ago2
