# Supplementary material for: A transient reversal of miRNA-mediated repression controls macrophage activation
Source: EMBO Rep. 2013 Sep 13;14(11):1008–16. doi: 10.1038/embor.2013.149 (PMC3851954; doi:10.1038/embor.2013.149)
Supplement: Source data for Figure 2 [file embor2013149df2.pdf]

# A transient reversal of miRNA-mediated repression controls macrophage activation

Anup Mazumder[a], Mainak Bose[a], Abhijit Chakraborty[b], Saikat Chakrabarti[b] and Suvendra N. Bhattacharyya[a,1]

[a]RNA Biology Research Laboratory, Molecular and Human Genetics Division, CSIR-Indian Institute of Chemical Biology,
Kolkata - 700032, India.
[b]Structural Biology and Bioinformatics Division, CSIR-Indian Institute of Chemical Biology, Kolkata - 700032, India.

[1]To whom correspondence should be addressed

Fig 2A

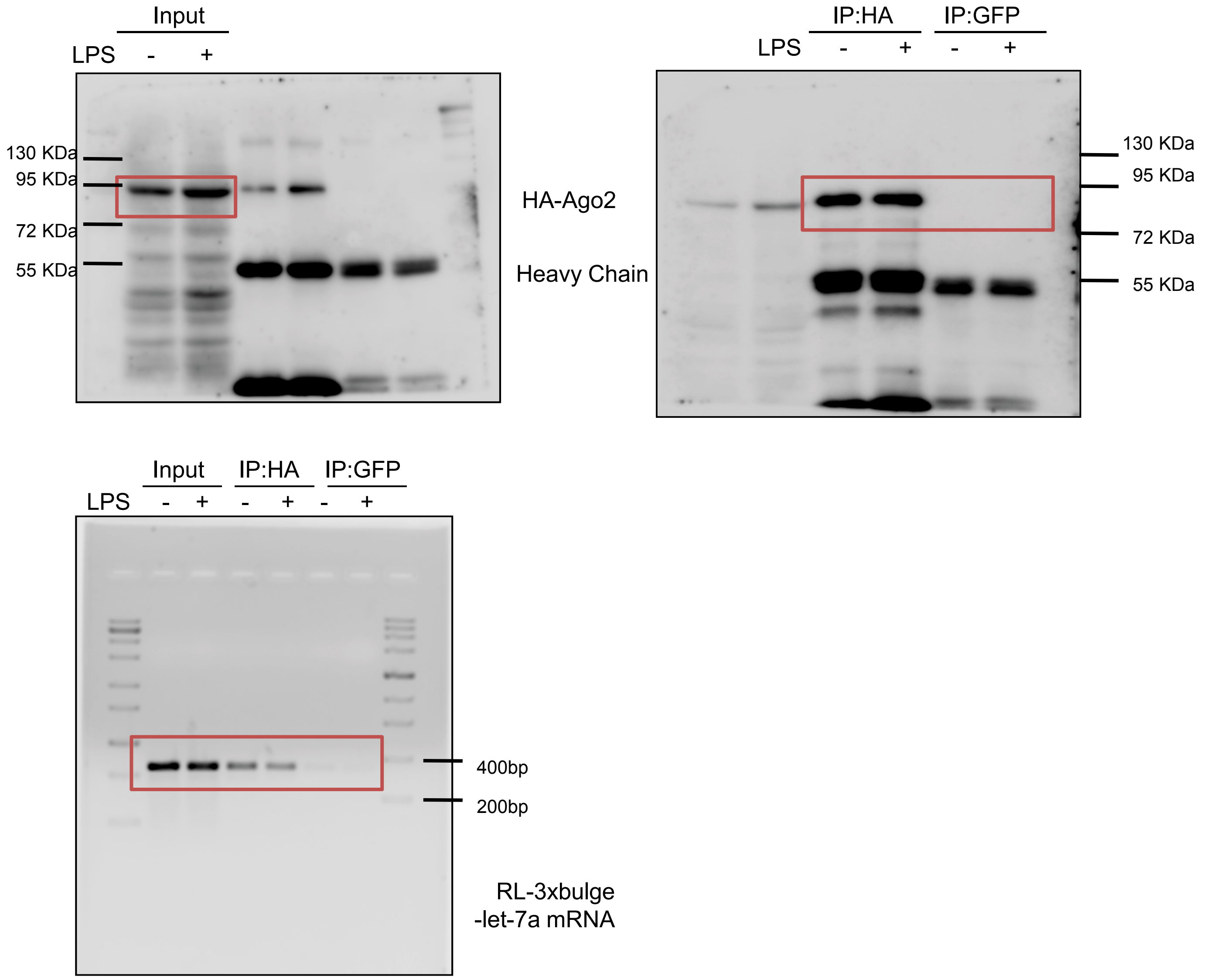
Input
LPS - +
130 KDa
95 KDa
72 KDa
55 KDa
HA-Ago2
Heavy Chain
IP:HA
IP:GFP
LPS - + - +
130 KDa
95 KDa
72 KDa
55 KDa
Input
IP:HA
IP:GFP
LPS - + - + - +
400bp
200bp
RL-3xbulge
-let-7a mRNA

Fig 2C

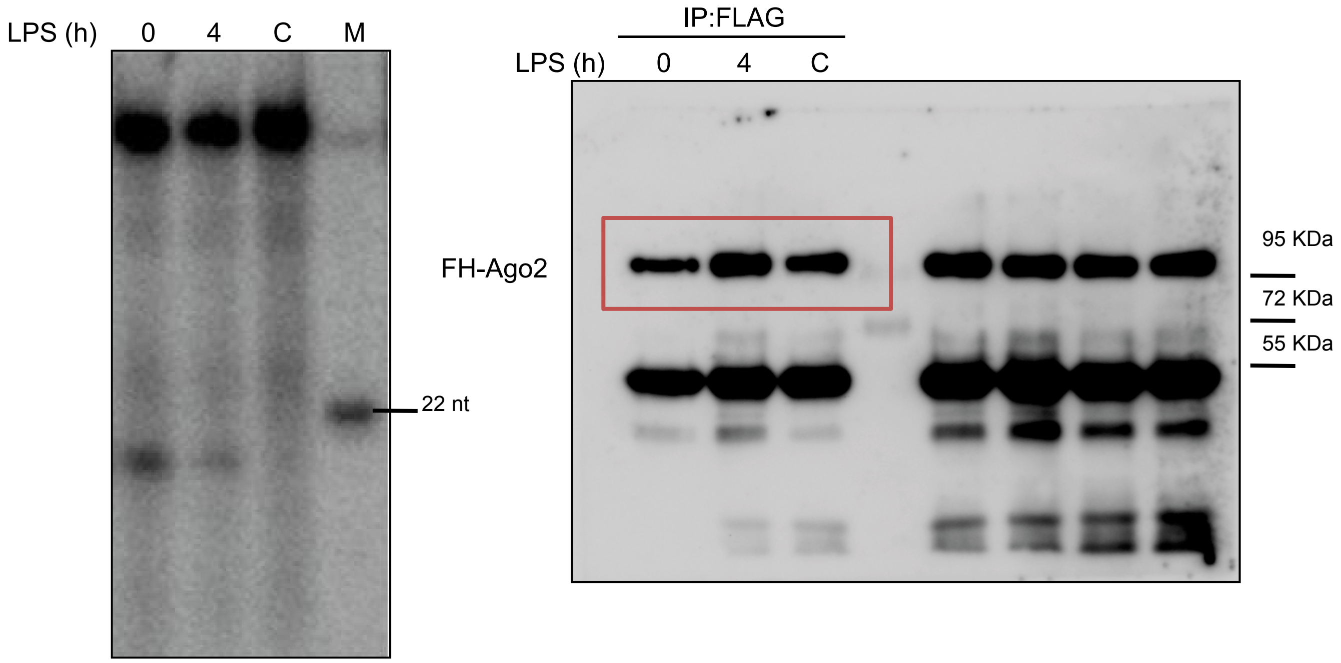
LPS (h) 0 4 C M
22 nt
FH-Ago2
IP:FLAG
LPS (h) 0 4 C
95 KDa
72 KDa
55 KDa

## Fig 2D

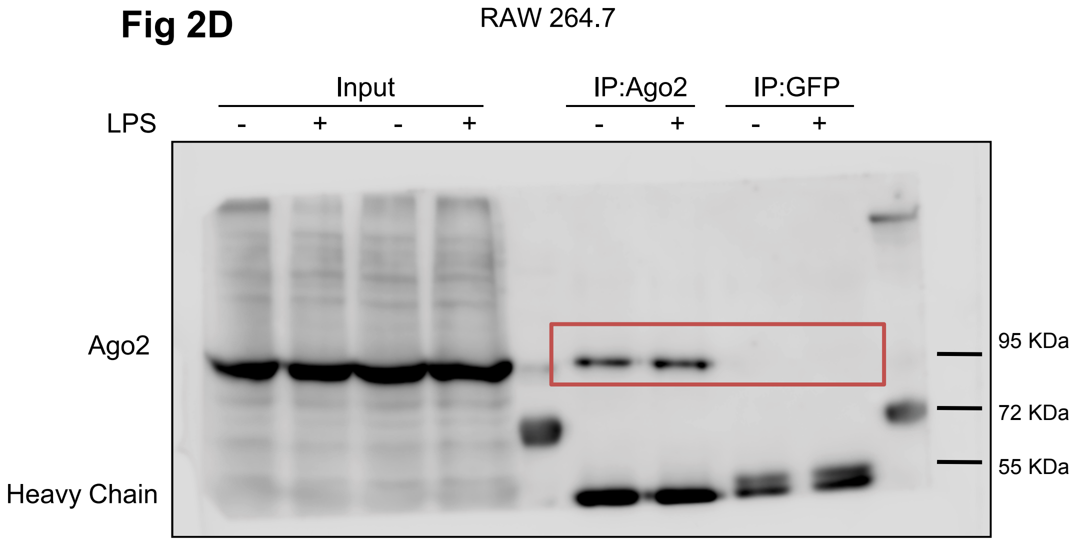
RAW 264.7
Input
IP:Ago2
IP:GFP
LPS
- + - + - + - +
Ago2
Heavy Chain
95 KDa
72 KDa
55 KDa

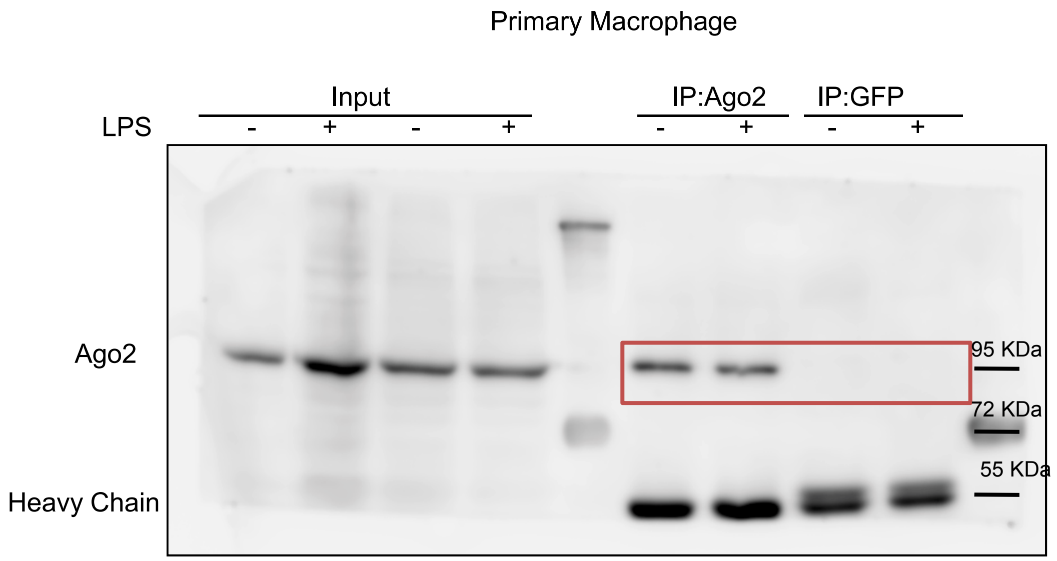
Primary Macrophage
Input
IP:Ago2
IP:GFP
LPS
- + - + - + - +
Ago2
Heavy Chain
95 KDa
72 KDa
55 KDa

## Fig 2E

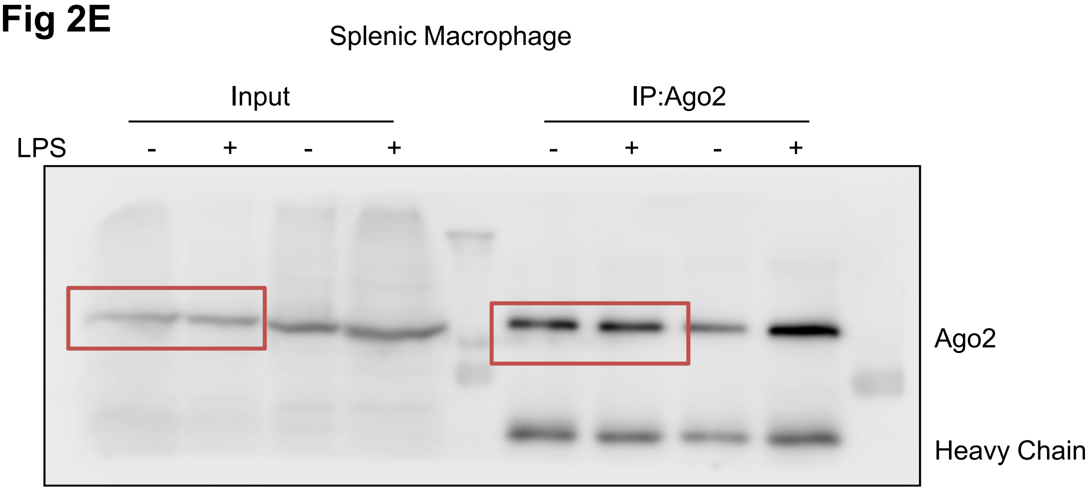
Splenic Macrophage
Input
IP:Ago2
LPS
- + - + - + - +
Ago2
Heavy Chain
