# Supplementary material for: A transient reversal of miRNA-mediated repression controls macrophage activation
Source: EMBO Rep. 2013 Sep 13;14(11):1008–16. doi: 10.1038/embor.2013.149 (PMC3851954; doi:10.1038/embor.2013.149)
Supplement: Source data for Figure 4 [file embor2013149df4.pdf]

A transient reversal of miRNA-mediated repression controls macrophage activation

Anup Mazumder[a], Mainak Bose[a], Abhijit Chakraborty[b], Saikat Chakrabarti[b] and Suvendra N. Bhattacharyya[a,1]

[a]RNA Biology Research Laboratory, Molecular and Human Genetics Division, CSIR-Indian Institute of Chemical Biology,
Kolkata - 700032, India.
[b]Structural Biology and Bioinformatics Division, CSIR-Indian Institute of Chemical Biology, Kolkata - 700032, India.

[1]To whom correspondence should be addressed

Fig 4A

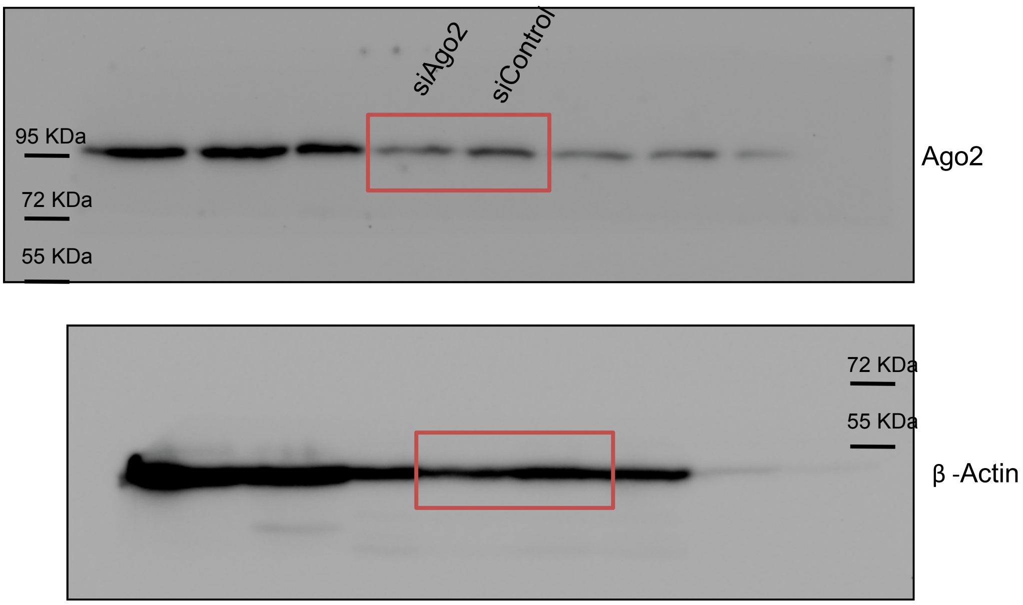

Fig 4E

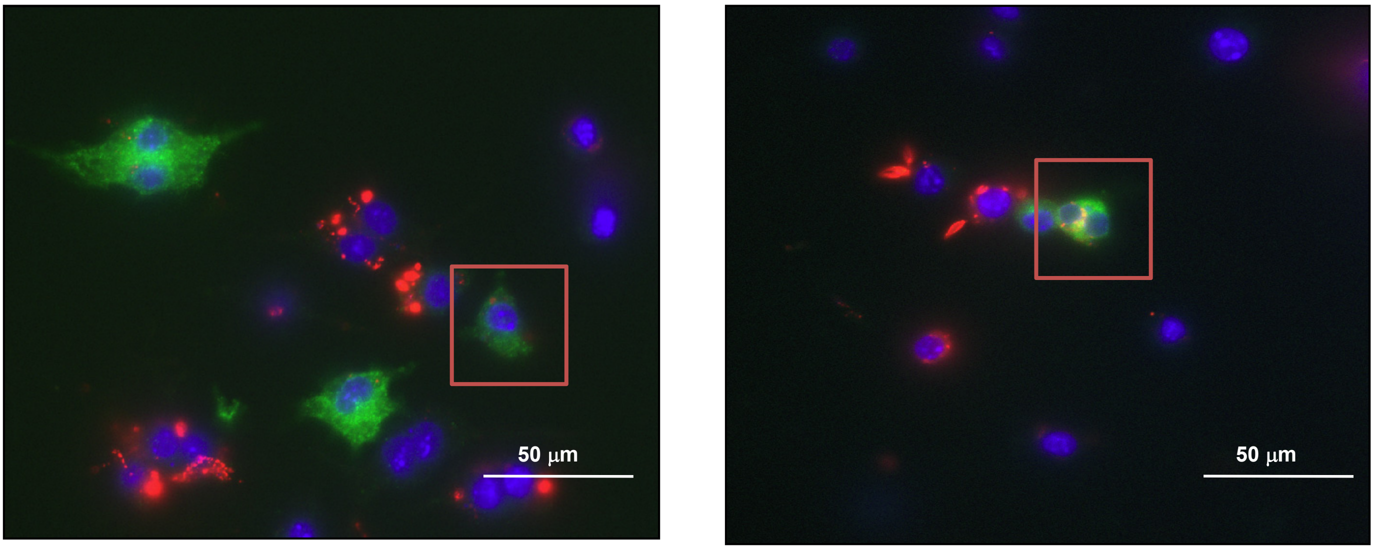

FH-Ago2

FH-Ago2 Y529F
